# Sex-specific association of ACAT-1 rs1044925 SNP and serum lipid levels in the hypercholesterolemic subjects

**DOI:** 10.1186/1476-511X-11-9

**Published:** 2012-01-13

**Authors:** Dong-Feng Wu, Rui-Xing Yin, Lynn Htet Htet Aung, Qing Li, Ting-Ting Yan, Xiao-Na Zeng, Ke-Ke Huang, Ping Huang, Jin-Zhen Wu, Shang-Ling Pan

**Affiliations:** 1 Department of Cardiology, Institute of Cardiovascular Diseases, the First Affiliated Hospital, Guangxi Medical University, 22 Shuangyong Road, Nanning 530021, Guangxi, People's Republic of China; 2 Department of Pathophysiology, School of Premedical Sciences, Guangxi Medical University, Nanning 530021, Guangxi, People's Republic of China

## Abstract

**Background:**

Acyl-CoA:cholesterol acyltransferase (ACAT) is a key enzyme in cellular cholesterol homeostasis and in atherosclerosis. The cellular cholesterol efflux correlated with serum high-density lipoprotein cholesterol (HDL-C) concentrations has shown to be impaired in hyperlipidemic mice. The present study was carried out to clarify the association of ACAT-1 rs1044925 single nucleotide polymorphism (SNP) and serum lipid levels in the hyperlipidemic subjects.

**Methods:**

A total of 821 unrelated subjects (hyperlipidemia, 476; normolipidemia, 345) aged 15-80 were included in the study. Genotyping of the ACAT-1 rs1044925 SNP was performed by polymerase chain reaction and restriction fragment length polymorphism combined with gel electrophoresis, and then confirmed by direct sequencing.

**Results:**

There was no significant difference in the genotypic and allelic frequencies of ACAT-1 rs1044925 SNP between the normolipidemic and hyperlipidemic subjects. The levels of total cholesterol (TC), HDL-C and apolipoprotein (Apo) AI in hyperlipidemic subjects were different between the AA and AC/CC genotypes in male but not in female (*P *< 0.05-0.01), the C allele carriers had higher serum TC, HDL-C and ApoAI levels than the C allele noncarriers. The association of genotypes and serum HDL-C and ApoAI levels in hyperlipidemia was found mainly in the male subjects with hypercholesterolemia but not in those with hypertriglyceridemia. There were no significant differences in serum lipid levels between the AA and AC/CC genotypes in the normolipidemic subjects.

**Conclusions:**

The present study shows that the C allele carriers of ACAT-1 rs1044925 SNP in male hyperlipidemic subjects had higher serum TC, HDL-C and ApoAI levels than the C allele noncarriers. There is a sex (male)-specific association of ACAT-1 rs1044925 SNP and serum HDL-C and ApoAI levels in the hypercholesterolemic subjects.

## Introduction

Cholesterol is present in tissues and in plasma lipoproteins either as free cholesterol or cholesteryl esters. Cellular cholesterol exists both as a free sterol and as esterified cholesterol. Acyl-coenzyme A: cholesterol acyltransferase (ACAT) is an intracellular enzyme that biosynthesizes cholesteryl esters from long-chain fatty acyl-CoA and cholesterol in various tissues [1], playing a major role in cellular cholesterol homeostasis. ACAT has been found to be present as two isoforms, ACAT-1 and ACAT-2, with different intracellular localizations, membrane topology in mammalian species, and metabolic function for each enzyme [2-4]. ACAT-1 is ubiquitously expressed in various tissues and cells including brain, adrenal glands, kidneys [5-7], and macrophages [8] and is responsible for foam cell formation in macrophages, whereas ACAT-2 is expressed only in intestine and liver [2,3,9] and is in charge of the cholesterol absorption process in intestinal mucosal cells [10].

ACATs have been considered as a target to develop novel therapeutic agents to control hypercholesterolemia, atherosclerosis, and Alzheimer's disease [11] because its potential role in cholesterol metabolism and atherosclerosis. The inhibition of ACAT activity has been associated with decreased plasma cholesterol levels by suppressing cholesterol absorption and by diminishing the assembly and secretion of apolipoprotein (Apo) B-containing lipoproteins such as very low density lipoprotein (VLDL). ACAT inhibition also prevents the conversion of macrophages into foam cells in the arterial walls, a critical event in the development of atherosclerosis [11,12]. However, whether ACAT inhibitors will serve as effective antiatherosclerosis drugs is currently under debate. In two recent researches, non-slection ACAT inhibition is not an effective strategy for limiting atherosclerosis, but may promote atherogenesis [13,14]. ACAT-1 deficiency did not prevent the development of atherosclerotic lesions either apoE-deficient or LDLR-deficient mice [15,16]. Studies showed that ACAT-1 deficiency is detrimental attribute to the cytotoxicity of accumulated free cholesterol caused by the impaired cellular cholesterol efflux in hyperlipidemic models [17,18]. Thus, a major effect of macrophage ACAT-1 depletion may be a disrupted of ATP-binding cassette transporter A1 (ABCA1)-mediated cholesterol efflux in the lipid-loaden condition [18]. Moreover, the extent of cholesterol efflux is highly correlated with serum ApoAI and high-density lipoprotein cholesterol (HDL-C) concentrations [19-21]. In a study of heterozygous subjects for ABCA1 mutations, the levels of cholesterol efflux account for 82% of the variation in HDL-C. Each 8% change in ABCA1-mediated efflux is predicted to be associated with a 0.1 mmol/L change in HDL-C [20]. In addition, previous studies have found the ACAT-1 gene polymorphisms affect serum HDL-C levels in endogenous hypertriglyceridemia [22], as well as hyperlipidemia [23]. Based on the above concept, we hypothesize that the ACAT-1 rs1044925 SNP might influence the cellular cholesterol efflux and regulate the concentration of serum HDL-C and ApoAI in the hyperlipidemic subjects. Therefore, the aim of the present research was to determine whether the ACAT-1 rs1044925 SNP affect the concentration of serum lipid levels associated with the cellular cholesterol efflux in the hyperlipidemic subjects.

## Materials and methods

### Study population

A total of 476 Han Chinese subjects of hyperlipidemia and 345 Han Chinese subjects of normolipidemia were randomly selected from our previous stratified randomized cluster samples [24,25]. The individuals with TC > 5.17 mmol/L and/or triglyceride (TG) > 1.70 mmol/L were defined as hyperlipidemica, hyperlipidemica was divided into (type IIa) hypercholesterolemia (TC > 5.17 mmol/L and TG ≤ 1.70 mmol/L) and (types IIb, IV and V) hypertriglyceridemia (TG > 1.70 mmol/L). The individuals with TC ≤ 5.17 mmol/L and TG ≤ 1.70 mmol/L were defined as normolipidemic. All study subjects had no evidence of any chronic illness, including hepatic, renal, or thyroid. The participants with a history of heart attack or myocardial infarction, stroke, congestive heart failure, diabetes or fasting blood glucose ≥ 7.0 mmol/L determined by glucose meter have been excluded. The participants were not taking medications known to affect serum lipid levels. The present study was approved by the Ethics Committee of the First Affiliated Hospital, Guangxi Medical University. Informed consent was obtained from all subjects.

### Epidemiological survey

The survey was carried out using internationally standardized methods [26]. Information on demographics, socioeconomic status, and lifestyle factors was collected with standardized questionnaires. The alcohol information included questions about the number of liangs (about 50 g) of rice wine, corn wine, rum, beer, or liquor consumed during the preceding 12 months. Alcohol consumption was categorized into groups of grams of alcohol per day: < 25 and ≥ 25. Smoking status was categorized into groups of cigarettes per day: < 20 and ≥ 20. Sitting blood pressure was measured using a mercury sphygmomanometer on 3 separated intervals after the subjects had a 5-minute rest, and the average of the three measurements was used for the level of blood pressure. Body mass index (BMI) was calculated as weight in kg divided by the square of height in meters (kg/m^2^).

### Biochemical analysis

Venous blood samples were obtained from all subjects after at least 12 hours of fasting. The levels of serum TC, TG, HDL-C, and low-density lipoprotein cholesterol (LDL-C) in samples were determined by enzymatic methods with commercially available kits. Serum ApoAI and ApoB levels were detected by the immunoturbidimetric immunoassay using a commercial kit [24,25].

### DNA amplification and genotyping

Genomic DNA was isolated from peripheral blood leukocytes using the phenol-chloroform method [27]. Genotyping of the ACAT-1 rs1044925 SNP was performed by polymerase chain reaction and restriction fragment length polymorphism (PCR-RFLP) [22,27,28]. PCR amplification was performed using 5'-TATATTAAGGGGATCAGAAGT-3' and 5'-CCACCTAAAAACATACTACC-3' (Sangon, Shanghai, People's Republic of China) as the forward and reverse primer pairs; respectively. After *Rsa *I restriction enzyme digestion of the amplified DNA, genotypes were identified by electrophoresis on 1.5% agarose gels and visualized with ethidium-bromide staining ultraviolet illumination. Genotypes were scored by an experienced reader blinded to epidemiological data and serum lipid levels. Six samples (AA, AC and CC genotypes in two; respectively) detected by the PCR-RFLP were also confirmed by direct sequencing.

### Statistical analyses

All statistical analyses were done with the statistical software package SPSS 13.0 (SPSS Inc., Chicago, Illinois). Quantitative variables were expressed as mean ± standard deviation (serum TG levels were presented as medians and interquartile ranges). Qualitative variables were expressed as percentages. Allele frequency was determined via direct counting, and the standard goodness-of-fit test was used to test the Hardy-Weinberg equilibrium. Difference in genotype distribution between the groups was obtained using the chi-square test. The difference in general characteristics between normolipidemic and hyperlipidemic subjects was tested by the Student's unpaired *t*-test. The association of genotypes and serum lipid parameters was tested by analysis of covariance (ANCOVA). Sex, age, BMI, blood pressure, alcohol consumption, cigarette smoking were adjusted for the statistical analysis. A *P *value of less than 0.05 was considered statistically significant.

## Results

### General characteristics and serum lipid levels

Table 1 gives the general characteristics and serum lipid levels between the normolipidemic and hyperlipidemic subjects. The levels of BMI, systolic blood pressure, diastolic blood pressure, serum TC, TG, HDL-C, LDL-C, ApoAI and ApoB were lower, and the ratio of ApoAI to ApoB were higher in normolipidemic than in hyperlipidemic subjects (*P *< 0.05-0.001). The percentage of subjects who consumed alcohol < 25 g/day was higher and the percentage of subjects who consumed alcohol ≥ 25 g/day was lower in normolipidemia than in hyperlipidemia (*P *< 0.01). There was no significant difference in the levels of pulse pressure, age structure, the percentage of subjects who smoked cigarettes, or the ratio of male to female between the two groups.

**Table 1:** The general characteristics and serum lipid levels between the normolipidemic an hyperlipidemic subjects

Parameter	Normolipidemia	Hyperlipidemia	*t *(χ^2^)	*P*
Number	345	476	-	-
Male/female	154/191	224/252	0.472	0.492
Age (years)	42.50 ± 14.91	43.82 ± 15.26	-1.240	0.215
Body mass index (kg/m^2^)	22.30 ± 2.63	23.34 ± 3.36	-4.954	0.000
Systolic blood pressure (mmHg)	120.85 ± 15.84	124.74 ± 17.37	-3.331	0.001
Diastolic blood pressure (mmHg)	75.30 ± 9.99	78.98 ± 10.73	-5.045	0.000
Pulse pressure (mmHg)	45.57 ± 11.67	45.76 ± 11.81	-0.220	0.826
Cigarette smoking [n (%)]				
Nonsmoker	242(70.1)	336(70.6)		
< 20 cigarettes/day	37(10.7)	36(7.6)	2.968	0.227
≥20 cigarettes/day	66(19.1)	104(21.8)		
Alcohol consumption [n (%)]				
Nondrinker	206(59.7)	290(60.9)		
< 25 g/day	119(34.5)	130(27.3)	11.146	0.004
≥ 25 g/day	20(5.8)	56(11.8)		
Total cholesterol (mmol/L)	4.31 ± 0.59	5.60 ± 0.96	-23.796	0.000
Triglyceride (mmol/L)	0.89(0.42)	1.59(1.11)	-14.552	0.000
HDL-C (mmol/L)	1.89 ± 0.41	1.97 ± 0.58	-2.035	0.042
LDL-C (mmol/L)	2.37 ± 0.49	3.15 ± 0.87	-16.334	0.000
Apolipoprotein (Apo) AI (g/L)	1.41 ± 0.22	1.50 ± 0.31	-4.857	0.000
ApoB (g/L)	0.82 ± 0.15	1.05 ± 0.23	-16.887	0.000
ApoAI/ApoB	1.79 ± 0.56	1.51 ± 0.58	6.762	0.000

HDL-C, high-density lipoprotein cholesterol; LDL-C, low-density lipoprotein cholesterol. The value of triglyceride was presented as median (interquartile range), the difference between the two groups was determined by the Wilcoxon-Mann-Whitney test.

### Genotypic and allelic frequencies

The frequency of ACAT-1 alleles and genotypes is shown in Table 2. The frequency of A and C alleles was 86.7% and 13.3% in normolipidemia, and 88.0% and 12.0% in hyperlipidemia (*P *> 0.05); respectively. The frequency of AA, AC and CC genotypes was 74.8%, 23.8% and 1.4% in normolipidemia, and 77.3%, 21.4% and 1.3% in hyperlipidemia (*P *> 0.05); respectively. There was also no significant difference in the genotypic and allelic frequencies between males and females in both groups.

**Table 2:** The genotype and allele frequencies of ACAT1 rs1044925 SNP between the normolipidemic and hyperlipidemic subjects [n (%)]

Group	n	Genotype			Allele	
			
		AA	AC	CC	A	C
Normolipidemia	345	258 (74.8)	82 (23.8)	5 (1.4)	598 (86.7)	92 (13.3)
Hyperlipidemia	476	368 (77.3)	102 (21.4)	6 (1.3)	838 (88.0)	114 (12.0)
χ^2^	-	0.709			0.673	
*P*	-	0.701			0.412	
Normolipidemia						
Male	154	111 (72.1)	42 (27.3)	1 (0.6)	264 (85.7)	44 (14.3)
Female	191	147 (77.0)	40 (20.9)	4 (2.1)	334 (87.4)	48 (12.6)
χ^2^	-	2.938			0.437	
*P*	-	0.230			0.509	
Hyperlipidemia						
Male	224	168 (75.0)	52 (23.2)	4 (1.8)	388 (86.6)	60 (13.4)
Female	252	200 (79.4)	50 (19.8)	2 (0.8)	450 (89.3)	54 (10.7)
χ^2^	-	1.848			1.614	
*P*	-	0.397			0.204	

### Genotypes and serum lipid levels

As shown in Table 3, the levels of TC, HDL-C and ApoAI in hyperlipidemia but not in normolipidemia were different between the AA and AC/CC genotypes in males but not in females (*P *< 0.05 for all). The subjects with AC/CC genotype had higher serum TC, HDL-C and ApoAI levels than the subjects with AA genotype. There was no difference in serum lipid parameters between the AA and AC/CC genotypes in normolipidemia (*P *> 0.05 for all). When hyperlipidemia was divided into hypercholesterolemia and hypertriglyceridemia, we found that the levels of HDL-C and ApoAI in hypercholesterolemia but not in hypertriglyceridemia were different between the AA and AC/CC genotypes in males but not in females (Table 4), the subjects with AC/CC genotype had higher serum HDL-C and ApoAI levels than the subjects with AA genotype. In addition, we also found that the levels of TC in male hypercholesterolemia or hypertriglyceridemia were not different between the AA and AC/CC genotypes. But the levels of TC in female hypercholesterolemia but not in female hypertriglyceridemia were lower in AC/CC genotype than in AA genotype (*P *< 0.05).

**Table 3:** The genotypes of ACAT-1 rs1044925 SNP and serum lipid levels between the normolipidemic and hyperlipidemic subjects

Genotype	n	TC (mmol/L)	TG (mmol/L)	HDL-C (mmol/L)	LDL-C (mmol/L)	ApoAI (g/L)	ApoB (g/L)	ApoAI/ ApoB
Normolipidemia								
AA	258	4.30 ± 0.61	0.89(0.42)	1.90 ± 0.41	2.37 ± 0.49	1.41 ± 0.22	0.82 ± 0.15	1.79 ± 0.59
AC/CC	87	4.34 ± 0.50	0.92(0.48)	1.87 ± 0.42	2.37 ± 0.50	1.41 ± 0.22	0.82 ± 0.14	1.77 ± 0.49
*F*	-	0.201	-1.034	0.323	0.009	0.009	0.000	0.083
*P*	-	0.654	0.301	0.570	0.926	0.927	0.994	0.773
Male								
AA	111	4.25 ± 0.73	0.90(0.42)	1.86 ± 0.42	2.37 ± 0.54	1.38 ± 0.22	0.83 ± 0.17	1.76 ± 0.68
AC/CC	43	4.27 ± 0.48	0.89(0.37)	1.80 ± 0.47	2.33 ± 0.48	1.35 ± 0.21	0.83 ± 0.13	1.67 ± 0.41
*F*	-	0.029	-0.173	0.608	0.171	0.579	0.000	0.662
*P*	-	0.866	0.862	0.437	0.680	0.448	0.988	0.417
Female								
AA	147	4.34 ± 0.51	0.88(0.41)	1.94 **± **0.41	2.36 ± 0.46	1.43 ± 0.21	0.82 ± 0.14	1.82 ± 0.51
AC/CC	44	4.43 ± 0.52	0.96(0.50)	1.94 **± **0.35	2.45 ± 0.51	1.46 ± 0.22	0.82 ± 0.15	1.86 ± 0.55
*F*	-	1.062	-1.293	0.000	1.067	0.667	0.034	0.234
*P*	-	0.304	0.196	0.996	0.303	0.415	0.854	0.629
Hyperlipidemia								
AA	368	5.57 ± 0.96	1.60(1.12)	1.95 ± 0.57	3.15 ± 0.87	1.49 ± 0.30	1.04 ± 0.22	1.51 ± 0.60
AC/CC	108	5.72 ± 0.96	1.53(1.08)	2.04 ± 0.64	3.15 ± 0.84	1.53 ± 0.34	1.06 ± 0.24	1.51 ± 0.49
*F*	-	2.314	-0.630	2.401	0.006	1.465	0.389	0.000
*P*	-	0.129	0.529	0.122	0.940	0.227	0.533	0.995
Male								
AA	168	5.48 ± 0.92	1.78(1.51)	1.87 ± 0.53	3.06 ± 0.89	1.48 ± 0.30	1.02 ± 0.24	1.59 ± 0.79
AC/CC	56	5.84 ± 1.03	1.75(2.04)	2.10 ± 0.74	3.13 ± 0.99	1.58 ± 0.37	1.03 ± 0.27	1.63 ± 0.59
*F*	-	5.911	1.005	9.060	0.309	5.382	0.192	0.135
*P*	-	0.016	0.315	0.003	0.579	0.021	0.662	0.714
Female								
AA	200	5.63 ± 0.98	1.50(1.03)	2.01 ± 0.59	3.23 ± 0.85	1.50 ± 0.30	1.06 ± 0.20	1.45 ± 0.37
AC/CC	52	5.61 ± 0.87	1.48(0.99)	1.96 ± 0.51	3.17 ± 0.64	1.46 ± 0.29	1.09 ± 0.21	1.38 ± 0.31
*F*	-	0.028	-0.081	0.312	0.286	0.803	0.521	1.912
*P*	-	0.868	0.935	0.577	0.593	0.371	0.471	0.168

TC, total cholesterol; TG, triglyceride; HDL-C, high-density lipoprotein cholesterol; LDL-C, low-density lipoprotein cholesterol; ApoAI, apolipoprotein AI; ApoB, apolipoprotein B

**Table 4:** The genotypes of ACAT-1 rs1044925 SNP and serum lipid levels between the hypercholesterolemic and hypertriglycemic subjects

Hypercholesterolemia					Hypertriglycemia				
	
Genotype	n	TC (mmol/L)	HDL-C (mmol/L)	ApoAI (g/L)	Genotype	n	TC (mmol/L)	HDL-C (mmol/L)	ApoAI (g/L)
Male					Male				
AA	76	5.79 ± 0.53	2.20 ± 0.53	1.55 ± 0.27	AA	92	5.25 ± 1.10	1.62 ± 0.41	1.41 ± 0.32
AC/CC	26	5.97 ± 0.70	2.49 ± 0.66	1.76 ± 0.37	AC/CC	30	5.66 ± 1.23	1.70 ± 0.41	1.43 ± 0.25
*F*	-	1.785	5.742	9.200	*F*	-	2.905	0.998	0.053
*P*	-	0.185	0.019	0.003	*P*	-	0.091	0.320	0.819
Female					Female				
AA	118	5.92 ± 0.84	2.27 ± 0.51	1.60 ± 0.31	AA	82	5.23 ± 1.04	1.64 ± 0.49	1.37 ± 0.24
AC/CC	36	5.62 ± 0.39	2.14 ± 0.38	1.52 ± 0.25	AC/CC	16	5.55 ± 1.48	1.45 ± 0.47	1.31 ± 0.29
*F*	-	4.406	1.890	1.762	*F*	-	1.435	2.273	0.934
*P*	-	0.046	0.171	0.186	*P*	-	0.234	0.135	0.336

## Discussion

In the present study, we showed that the levels of HDL-C, LDL-C, ApoAI and ApoB were higher in subjects with hyperlipidemia than in subjects with normolipidemia besides serum TC and TG levels, whereas the ratio of ApoAI to ApoB was lower in subjects with hyperlipidemia than in subjects with normolipidemia. Although the evidence suggests the inverse relation of serum HDL-C and TG levels [29], the mechanism of this association is still unclear. In several our previous studies [24,25], elevated serum HDL-C and TG levels was found consistently in the hypertensives, drinkers and obese subjects. It is well known that dyslipidemia is a multifactorial origin, including hereditary and acquired risk factors.

The genotypic and allelic frequencies of ACAT-1 rs1044925 SNP in diverse populations are inconsistent. Zhao *et al. *[28] reported that there was not different in the minor allele frequency between normal controls (9.7%) and Alzheimer's disease patients (9.3%). In another recent study, Li *et al. *[22] also found no significant difference in the C allele frequency between normal controls (13.7%) and endogenous hypertriglyceridemia patients (15.3%). However, the C allele frequency of ACAT-1 rs1044925 SNP was very high in the population of central and Southern Europe (35.4%) [30]. In the current study, we showed that there were no significant differences in the genotypic and allelic frequencies of ACAT-1 rs1044925 SNP between normolipidemia and hyperlipidemia (13.3% vs. 12.0%, *P *> 0.05), or between males and females in both groups. These results indicate that the ACAT-1 rs1044925 SNP may have a racial/ethnic, or disease specificity.

In the present study, the associations of ACAT-1 rs1044925 SNP and serum lipid levels were determined according to the subjects with or without hyperlipidemia. We found that the effect of ACAT-1 rs1044925 SNP on serum TC, HDL-C and ApoAI was more prominent in subjects with hyperlipidemia than in subjects with normolipidemia. There is also a sex (male)-specific association of ACAT-1 rs1044925 SNP and serum HDL-C and ApoAI levels in subjects with hypercholesterolemia but not in subjects with hypertriglyceridemia. In a previous study, Ohta *et al. *[23] have found that the -77G > A variant affected serum HDL-C and ApoAI concentriations in hyperlipidemic subjects. The concentration of plasma HDL-C and ApoAI were significantly higher in A allele carriers than in A allele noncarriers. This is consistent with our results in the ACAT-1 rs1044925 SNP. In the current study, however, we found that the associations of ACAT-1 rs1044925 SNP and serum HDL-C and ApoAI levels were only in the hypercholesterolemic subjects. To our knowledge, these findings have not been previously explored. It is unknown whether the ACAT-1 variants influence the ACAT1 activity. Ohta *et al. *[23] have shown a relation between the -77G > A mutation and the plasma HDL concentration in hyperlipidemic subjects. It is likely that ACAT-1 gene with the mutation may not provide enough ACAT-1 protein for efficient catalysis of increased intracellular free cholesterol in the lipid-loaden status. The increased free cholesterol will increase the efflux of cholesterol as well as increase the concentration of serum HDL-C and ApoAI. In another study of the association of ACAT-1 rs1044925 SNP and plasma lipid levels, however, Li *et al. *[22] found that serum HDL-C levels in patients with endogenous hypertriglyceridemia in China were lower in the C allele carriers than in C allele noncarriers. These findings were opposite with our results in the male hypercholesterolemic but not in hypertriglyceridemic subjects.

In the present study, we showed that the C variant of ACAT-1 rs1044925 SNP could increase serum HDL-C and ApoAI levels in the male subjects with hypercholesterolemia, which may play a protective factor for atherosclerosis. In lipid-loaden macrophages, however, it was confirmed that ACAT-1 depletion in macrophages is proatherogenic [15,16]. To explain these conflicts, the possible reasons might as follows: Firstly, in the ACAT-1 deletion modol, the ABCA1-mediated cholesterol efflux to protect macrophages from free cholesterol toxicity may be not sufficient in the status of completely lack of ACAT-1 [23]. While in our study, the ACAT-1 rs1044925 SNP did not affect the ACAT-1 activity greatly. The pathways of the ABCA1-mediated cholesterol efflux may be sufficient in the physiological status. If there are some defects in the pathways of the ABCA1-mediated cholesterol efflux, the efflux of the cholesterol will decrease and the free cholesterol will increase in the cell. Thus, the rs1044925 C variant with the gene variants in pathways of the cholesterol efflux could be at higher risk for atherosclerosis. Secondly, ACAT-1 is ubiquitously expressed in various tissues and cells including adrenal glands, kidney and macrophages. Not only from macrophages, the circulating HDL-C levels were regulated by the cholesterol efflux from these cells that the ACAT-1 expressed in, therefore, the circulating HDL-C levels might reflect the cholesterol efflux from the others cell and tissues. Furthermore, although epidemiological studies demonstrated an inverse relationship between HDL-C levels and coronary heart disease, the serum HDL-C levels in part but do not always reflect cholesterol efflux capacity, a key metric of HDL function [31]. In the present study, we did not determine the association of ACAT-1 rs1044925 SNP and the level of cholesterol efflux, HDL particle sizes, as well as the extent of atherosclerosis. The only result directly present in our study was that the ACAT-1 rs1044925 SNP influenced the HDL-C and ApoAI levels in the male with hyperlipidemia, which suggests that the ACAT-1 rs1044925 SNP might influence the cellular cholesterol efflux in the male hyperlipidemic subjects.

In addition, we also found that serum TC levels were higher in AC/CC than in AA genotypes in the male hyperlipidemic subjects, but the levels of TC in male hypercholesterolemia or hypertriglyceridemia were not different between the AA and AC/CC genotypes. The levels of TC in female hypercholesterolemia but not in female hypertriglyceridemia were lower in AC/CC genotype than in AA genotype. The reason for this discrepancy is unclear. In ACAT-1 deficiency macrophages, cholesterol synthesis was increased by 134% in ACAT1 (-/-) macrophages compared to wildtype macrophages, which suggests that ACAT-1 affects the regulation of cholesterol metabolism in macrophages [32]. Therefore, the ACAT-1 rs1044925C variant had a higher TC levels may attribute to the C variant of the rs1044925 SNP could not produce enough ACAT-1 protein and induce an increased cholesterol synthesis in the macrophages.

## Conclusion

The present study shows that serum HDL-C and ApoAI levels in the male subjects with hypercholesterolemia but not in those with hypertriglyceridemia were significantly different between the AA and AC/CC genotypes. These results suggest that there is a sex (male)-specific association of ACAT-1 rs1044925 SNP and serum HDL-C and ApoAI levels in the hypercholesterolemic subjects. The ACAT-1 rs1044925 SNP might influence the cellular cholesterol efflux, and indirectly modulate serum HDL-C concentrations.

## Competing interests

The authors declare that they have no competing interests.

## Authors' contributions

DFW participated in the design, undertook genotyping, and drafted the manuscript. RXY conceived the study, participated in the design, carried out the epidemiological survey, collected the samples, and helped to draft the manuscript. LHHA, QL, TTY, XNZ, KKH and PH collaborated to the genotyping. JZW and SLP carried out the epidemiological survey and collected the samples. All authors read and approved the final manuscript.
